# Traditional Chinese Medical Care and Incidence of Stroke in Elderly Patients Treated with Antidiabetic Medications

**DOI:** 10.3390/ijerph15061267

**Published:** 2018-06-15

**Authors:** Yun-Wen Chiao, Yu-Jen Chen, Yu-Hsien Kuo, Chung-Yen Lu

**Affiliations:** 1 Department of Chinese Medicine, Dalin Tzu Chi Hospital, The Buddhist Tzu Chi Medical Foundation, Chiayi 62247, Taiwan; dl20658@tzuchi.com.tw; 2 Department of Chinese Medicine, Taipei City Hospital Zhongxing Branch, Taipei 10341, Taiwan; u100030029@cmu.edu.tw; 3 Department of Chinese Medicine, China Medical University Hospital, Taichung 40447, Taiwan; u100030033@cmu.edu.tw; 4 Department of Sport and Health Management, Da-Yeh University, Changhua 51591, Taiwan; 5 Department of Chinese Medicine, Show Chwan Memorial Hospital, Changhua 50008, Taiwan

**Keywords:** diabetes, stroke, traditional Chinese medicine

## Abstract

Objectives: Experimental research has shown that herbal and traditional Chinese medicines (TCM) may serve as complements to Western medicine treatments in the control of blood glucose and cardiovascular complications, but population-based studies are limited. We investigated the association between TCM use and subsequent risk of stroke in older patients with diabetes. Study design: The database used in this cohort study contained longitudinal medical claims for one million subjects randomly selected among beneficiaries of a universal health care program in Taiwan. We identified a cohort of patients with diabetes aged 65 years and older who initiated anti-diabetic medications from 2000 to 2012. Patients who had at least two TCM outpatient visits after their initiation of anti-diabetic medications were considered TCM users. Main outcome measures: The incidence of stroke was measured until 2013. Cox regression models with TCM use as a time-dependent variable were used to calculate the adjusted hazard ratios (HRs) comparing TCM use with no use. Results: Over the 13-year period, 17,015 patients were identified; 4912 (28.9%) of them were TCM users. The incidence of stroke during the follow-up (per 1000 person-years) was 22.8 in TCM users and 25.7 in non-users. TCM users had an adjusted HR of 0.93 for the incidence of ischemic stroke (95% confidence interval [CI] 0.83, 1.04) and of 0.89 for developing hemorrhagic stroke (95% CI 0.66, 1.19), compared with non-users. Conclusions: In this study, in older patients receiving Western medicine treatments for diabetes, TCM use was not associated with an increased risk of developing ischemic stroke and hemorrhagic stroke.

## 1. Introduction

The number of adult people with diabetes was estimated to have doubled over the past three decades, reaching 347 million in 2008 [1]. With the increasing epidemic of diabetes, concern has risen about the management of diabetes and its complications. Diabetes is a well-established risk factor for stroke [2], and the risk may heighten in the presence of other coexisting risk factors such as hypertension [3]. Stroke can lead to long-term neurologic consequences requiring life-long care and is the second leading cause of global deaths [4]. Given that older people have a high prevalence of diabetes and often have multiple comorbidities [5] that could amplify their risk of stroke, the prevention of stroke is of particularly importance in the elderly with diabetes. Treatment strategies specific to older people with diabetes are needed because of the heterogeneity and complexity of health conditions in this patient group [6]. 

Traditional Chinese medicine (TCM) has been used for thousands of years for the treatment of diabetes in the Chinese medical system [7]. Clinical trials and in vivo and in vitro studies have demonstrated that TCM medications, applied alone or as complements to Western medicine treatments, had beneficial effects on glycemic control and cardiovascular complications [8,9,10,11]. Furthermore, epidemiological studies have revealed beneficial effects of TCM on the quality of life [12] and on depression among people aged 50 years or above [13], suggesting that TCM may be a good supplement to Western medicines for the elderly. A recent study has shown that a complementary TCM therapy was associated with a decreased risk of stroke in patient with diabetes [14]. However, that study did not report age-specific data and did not analyze ischemic and hemorrhagic stroke separately. We are unaware of any population-based studies providing data on the vascular outcomes of TCM care in older adults with diabetes. Using claims data from the Taiwan National Health Insurance program (NHI), we investigated the association between TCM care and the risk of developing stroke in older patients treated with Western antidiabetic drugs.

## 2. Materials and Methods

### 2.1. Data Source

NHI is a compulsory, government-run health insurance system that provided comprehensive healthcare coverage, including TCM outpatient care services, to 96% of the entire 23 million Taiwan’s residents by the end of 1996 [15] and to more than 99% of the population by 2011 [16]. We did a retrospective cohort study using the Longitudinal Heath Insurance Database (LHID) 2000 [17], a medical claims database that includes one million subjects randomly selected among people insured by the NHI during 1996–2000. The LHID has been widely used for research purposes and is representative of the general population in terms of age and sex distributions [17]. The dataset consisted of multiple claims files including expenditures and orders of ambulatory care and inpatient care, prescription drugs, and registry for beneficiaries, which together provide information about diagnoses and details of healthcare services utilization. All diagnostic codes of the claims are recorded according to the International Classification of Diseases, 9th Revision, Clinical Modification (ICD-9-CM). We linked these files anonymously through an encrypted personal identification number to create patient-level longitudinal healthcare records. The Institutional Review Board of the Chang Gung Memorial Hospital approved this study (104-0925B).

### 2.2. Study Population

**Figure 1:**
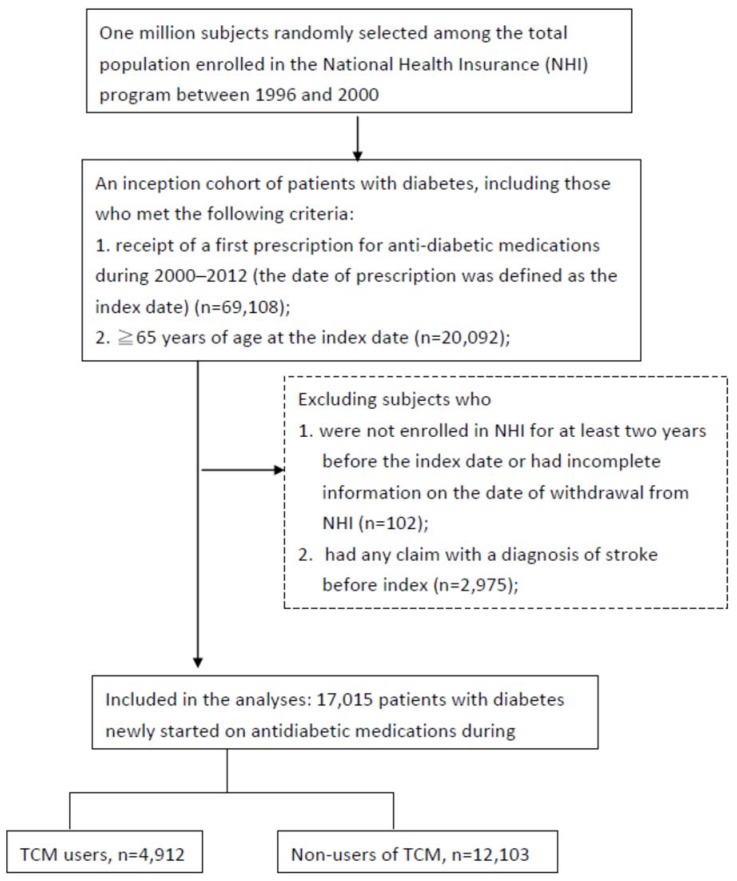
Flowchart of the selection of the study subjects. TCM: traditional Chinese medicine

Within the LHID population, we identified an inception cohort consisting of older patients who were new users of antidiabetic drugs. The cohort included subjects (i) who had a diagnosis of diabetes (ICD-9-CM code 250.xx) in any one inpatient claim or at least two outpatient claims >30 days apart within one year [18], (ii) who received a first prescription for any antidiabetic medication between 1 January 2000 and 31 December 2012, and (iii) who were aged 65 years and above on the date of the first prescription (the index date). We searched prescription claims from 1 January 1997 for each subject and used the earliest prescription after 2000, thereby ensuring that patients were new users without any prescription for antidiabetic drugs for at least three years before their index date. To provide a two-year medical history, we excluded subjects not enrolled in the NHI program at any time in the two years before their index date. Finally, patients were excluded if they had any inpatient or outpatient claim with a diagnosis of stroke (ICD-9-CM code 430.xx-438.xx) before their index date. The flowchart of the selection of the study subjects is shown in Figure 1.

### 2.3. Exposure to TCM

We defined TCM users as patients who had at least two claims for TCM outpatient visit on their index date or after and had received TCM treatments other than traumatology manipulative therapies, which are unlikely to be used for diabetes treatment. All the other patients in the diabetes cohort formed the group of non-users. TCM use was further classified into three groups. One group consisted of subjects who received acupuncture, one group consisted of those who received TCM treatments consisting of herbal formulas, and the third group consisted of patients who received both treatment modalities.

### 2.4. Patient Characteristics

The data on comorbidities were collected focusing on those known to be associated with an increased risk of stroke (Table 1). These comorbid conditions were considered to be present if the diagnosis codes of ICD-9-CM (Supplementary Materials Table S1) were recorded on at least one inpatient claim or two outpatient claims in the two years before the index date. We determined the number of outpatient visits to clinics of western medicine and TCM and the number of hospitalizations from claims spanning two years before the index date and during the follow-up period to depict the extent of health service use. The duration of the prescription of antidiabetic drugs was also ascertained by searching prescription datasets.

### 2.5. Outcome Variable

The study end point was stroke, defined by ICD-9-CM codes 430.xx-437.xx in the hospital discharge diagnosis. We further classified stroke into hemorrhagic stroke (ICD-9-CM codes 430.xx-432.xx) and ischemic stroke (ICD-9-CM codes 433.xx-437.xx). The follow-up duration started at the index date (i.e., the date of initiation of the antidiabetic treatment) and ended at the occurrence of stroke, withdrawal from the NHI program, or termination of this study on December 31, 2013, whichever came first. The enrollment rate in the NHI of the entire population was greater than 96% after 1996. Thus, the loss to follow-up of the study subjects was considered to be lower than 4%. Withdrawal from the NHI, which might be due to death or other reasons, was considered loss to follow-up and was treated as a censoring event. 

### 2.6. Statistical Analysis

The baseline characteristics of the TCM users and non-users were summarized using descriptive statistics. The differences in the baseline characteristics were examined using the *t* test for continuous variables and the χ^2^ test for categorical variables. To account for the immortal (or unexposed) time of TCM users [19], we assessed the association between TCM use and risk of developing stroke using Cox proportional hazard models with a time-dependent variable for TCM use. The TCM users and their follow-up person-time were classified into the non-users group until the date of their second visit to a TCM outpatient clinic and, thereafter, they were included in the group of TCM users. To control for confounding effects, we considered several comorbidities that were established risk factors of stroke and assessed whether these variables were associated with the risk of stroke in univariate analysis. We also assessed the number of prior hospital admissions, which has been shown to be related to geriatric conditions and worse general health [20,21], in an attempt to account for the potential indication bias. The final multivariable-adjusted models included age, sex, hypertension, hyperlipidemia, atrial fibrillation, ischemic heart disease, chronic kidney disease, and number of hospital admissions. The Cox models yielded the hazard ratio (HR) and the 95% confidence interval (CI) for stroke in TCM users, with non-users as the reference group. The estimated survival curves derived from a proportional hazard model with time-dependent covariates were also plotted to illustrate the probability of free-from-stroke over time. 

We repeated the Cox models in which the TCM use was further classified into use of acupuncture, use of herbal formulas, and use of both acupuncture and herbal formulas. To observe the effects of herbal formulas, we classified the herbal formulas by the way they act on the body and assessed their association with stroke using the multivariable-adjusted Cox regression. We also conducted a sensitivity analysis, in which the period of time between two claims for TCM outpatient visit was restricted to one year after the index date, to evaluate the robustness of the results. The data analyses were performed using SAS version 9.3 (SAS Institute Inc., Cary, NC, USA). All statistical tests were two-sided.

## 3. Results

The study cohort consisted of 17,015 new users of antidiabetic drugs, of whom 4912 (28.9%) had received TCM services after their first prescribed antidiabetic drugs (Figure 1). Table 1 presents the baseline characteristics of TCM users and non-users. The mean age of the TCM group and the non-user group was 72.0 (SD 5.2) years and 74.2 years (SD 6.7) (*p* < 0.001), respectively. TCM use was more common among women than men (57.0% vs. 50.0%, *p* < 0.001). The TCM users was less likely than the non-users to have cancer (2.7% vs. 4.6%, *p* < 0.001), atrial fibrillation (2.8% vs. 4.2%, *p* < 0.001), and chronic kidney disease (2.1% vs. 3.8%, *p* < 0.001), but more likely to have hyperlipidemia (28.2% vs. 23.8%, *p* < 0.001). The two groups were similar with respect to the prevalence of hypertension and ischemic heart disease. The TCM users had a greater number of visits to western medicine clinics but were less likely to have been admitted to hospital (*p* < 0.001 for both). The median follow-up duration was similar between the TCM users and non-users (3.9 years, respectively, *p* = 0.18). The median number of visits to TCM clinics during the follow-up period among the TCM users was 7 (the 25th and 75th percentile, 3 and 16, respectively).

Over the 13-year period, the incidences of stroke per 1000 person-years were 22.8 for the TCM users and 25.7 for the non-users, which yielded an unadjusted HR of 0.87 (95% CI, 0.79–0.97) (Table 2). After multivariable adjustments, the association weakened to a statistically non-significant level, with an adjusted HR of 0.92 (95% CI, 0.84–1.03). Similarly, we observed no association in the analyses in which the type of stroke was classified. The adjusted HR (95% CI) was 0.93 (0.83–1.04) for ischemic stroke and 0.89 (0.66–1.19) for hemorrhagic stroke. Sensitivity analyses did not change our essential finding that TCM use was not associated with the risk of developing ischemic stroke and hemorrhagic stroke (Supplementary Materials Table S2). The survival curves of time to all stroke, ischemic stroke, and hemorrhagic stroke for the TCM users and non-users are shown in Supplementary Materials Figure S1. 

When we assessed the risk of stroke according to the TCM treatments received by the patients, the patients who received acupuncture had a decreased risk of developing stroke (adjusted HR [95% CI], 0.77 [0.63–0.95]), compared with the non-users of TCM, whereas the use of herbal formulas or the combination of both treatments was not associated with the risk of stroke (adjusted HR [95% CI], 0.97 [0.86–1.08] and 1.19 [0.78–1.82], respectively) (Table 3). However, no association was observed for the three groups when stroke was classified into ischemic stroke and hemorrhagic stroke.

We performed a further analysis to examine the association between specific Chinese herbal formulas, classified by their actions on the body, and the risk of stroke (Table 4). We found no association between all categories of herbal formulas and the risk of ischemic stroke. However, an increased risk of hemorrhagic stroke was observed in association with wind-dampness-dispelling formulas (adjusted HR 1.80 [95% CI, 1.02–3.16], and phlegm-dispelling formulas (adjusted HR 2.02 [95% CI, 1.07–3.83]). 

## 4. Discussion

In this cohort study of new users of antidiabetic drugs aged 65 years and above, TCM use was not associated with a reduced incidence of ischemic stroke and hemorrhagic stroke. We also did not observe a significant association between TCM treatment modalities and both types of stroke. However, a formula-specific analysis showed that the use of dampness-dispelling formulas and phlegm-dispelling formulas was associated with an increased risk of developing hemorrhagic stroke.

The prevalence of TCM use in patients with diabetes may vary by age. An earlier study showed that in Taiwan, 78% of patients with diabetes ≥20 years of age had used TCM [22]. We found a much lower TCM using rate (28.9%) in the elderly patients with diabetes, which is lower than that reported in recent studies of older general populations in Taiwan. In a nationally representative panel survey, 47.8% of subjects aged 53 to 80 years reported their use of TCM [13]. Another study using claims data also found that 48% of subjects aged 65 year and older had ever used TCM [23]. These observations indicated that, among older people, TCM is not more often an option for patients with diabetes than for the general population. In addition, a lower prevalence rate of TCM use in our analysis might be explained by the differences in the definitions of TCM use. We required at least two visits in a TCM outpatient setting for an individual to be classified into the TCM use group.

One population-based study by Lee et al., which was also an analysis of NHI claims, reported the association between TCM use and subsequent risk of stroke [14]. Inconsistent with our observations, Lee et al. reported a reduced risk of stroke (all stroke) in association with TCM use. The discrepancies between ours and these findings might reflect the different methodologies used. Lee’s study was not specific to older patients and excluded subjects with several medical histories, including chronic kidney diseases, diabetic foot, trauma and fracture, because the authors evaluated multiple vascular complications. In addition, in that study, the TCM users were subjects who received TCM care for at least 30 days. Therefore, the study findings of our analysis and those of that study are likely to apply to different patient populations. 

There are strengths in our study. This large, representative population-based cohort study was less vulnerable to selection bias. The subjects in both the TCM group and the comparison group were new users of antidiabetic drugs. This design reduced the potential differences in diabetes duration and diabetes complications between the two groups. In addition, we used a Cox model with time-dependent variable for TCM initiation, which classified the stroke-free person-time of the TCM group before the TCM treatment as the unexposed (non-used) person-time, to avoid introducing immortal time bias.

Our findings must be interpreted in awareness of the potential limitations of this study. First, TCM services provided by healthcare institutions not contracted by NHI were not available in the claims database. The TCM use might be underestimated or misclassified to non-use if patients sought care outside the NHI-contracted network. However, the misclassification would involve a small portion of subjects, as 92% of TCM hospitals and clinics are contracted by the NHI [16]. Second, the use of Chinese herbal products might be underestimated because the claims data do not contain information on TCM drugs or products for which patients paid out of their own pocket. The TCM effect associated with stroke thus might be underestimated. Third, although we included several patients’ characteristics for multivariable adjustments, confounding by unmeasured factors or residual confounding might occur. In particular, the database does not contain lifestyle and behavior factors, such as diet, smoking, alcohol drinking, and exercise, which are risk factors of stroke [24]. A confounding bias might occur if these factors differ between the TCM users and the non-users. However, previous studies showed inconsistent results for the association between lifestyle and TCM use. In a study using data of the Taiwan National Health Interview Survey, which included subjects aged 18 years and above, a healthy lifestyle was associated with TCM use in men but not in women [25]. However, the Singapore Chinese Longitudinal Aging Study has suggested that among the elderly, smokers tended to use TCM, compared to non-smokers [26]. Further studies with complete information on the lifestyle variables leading to confounding assessments are needed to clarify the association between TCM therapy and incidence of stroke. Last, 98% of the NHI beneficiaries are Taiwanese residents, predominately ethnic Chinese. Thus, the results of this study might not be generalizable to other ethnic groups.

## 5. Conclusions

Among older adults who received Western antidiabetic treatments, those using TCM, compared with non-users of TCM, did not have a decreased risk of developing hemorrhagic stroke and ischemic stroke. Further studies are needed to evaluate the safety and effectiveness of specific Chinese herbal formulas, in particular dampness-dispelling formulas and phlegm-dispelling formulas, in the treatment of older patients with diabetes.

## Figures and Tables

**Table 1:** Baseline characteristics of patients with newly treated diabetes receiving or not TCM care.

Characteristic	Total	TCM Users	Non-Users	*p*-Value
	*n* = 17,015	*n* = 4912	*n* = 12,103	
Age, years				
Mean (SD)	73.6 (6.4)	72.0 (5.2)	74.2 (6.7)	<0.001
Women	8853 (52.0)	2801 (57.0)	6052 (50.0)	<0.001
Comorbidities at baseline *				
Hypertension	10,806 (63.6)	3066 (62.4)	7740 (64.0)	0.060
Hyperlipidemia	4263 (25.1)	1386 (28.2)	2877 (23.8)	<0.001
Atrial fibrillation	643 (3.8)	137 (2.8)	506 (4.2)	<0.001
Ischemic heart disease	4168 (24.5)	1227 (25.0)	2941 (24.3)	0.35
Cancer	687 (4.0)	132 (2.7)	555 (4.6)	<0.001
Chronic kidney disease	560 (3.3)	105 (2.1)	455 (3.8)	<0.001
Health care utilization at baseline *				
Number of visits to TCM outpatient clinics				
Mean (SD)	3.2 (8.7)	2.6 (5.4)	3.4 (9.7)	<0.001
Median (Q1, Q3)	0 (0, 2)	0 (0, 3)	0 (0, 2)	
Number of visits to western medicine outpatient clinics				
Mean (SD)	51.1 (36.8)	53.9 (35.8)	50.0 (37.1)	<0.001
Median (Q1, Q3)	43 (25, 67)	47 (29, 70)	42 (24, 66)	
Number of hospital admissions				
Mean (SD)	0.7 (1.5)	0.5 (1.0)	0.8 (1.6)	<0.001
Median (Q1, Q3)	0 (0, 1)	0 (0, 1)	0 (0, 1)	
0	11,080 (65.1)	3496 (71.2)	7584 (62.6)	<0.001
1	3402 (20.0)	905 (18.4)	2497 (20.7)	
≥2	2533 (14.9)	511 (10.4)	2022 (16.8)	
Duration of follow-up, years				
Mean (SD)	4.7 (3.4)	4.7 (3.3)	4.6 (3.4)	0.1835
Median (Q1, Q3)	4.0 (1.9, 6.9)	4.1 (1.9, 7.1)	3.9 (1.9, 6.9)	
Number of outpatient visits to TCM clinics during follow-up period				
Mean (SD)	-	15.2 (24.8)		
Median (Q1, Q3)	-	7 (3, 16)		

The values are presented as number of subjects and percentages unless otherwise indicated. Abbreviations: TCM, traditional Chinese medicine; Q1, first quartile; Q3, third quartile. * Defined by searching claims within two years before the index date.

**Table 2:** Incidence rate and hazard ratio of incidence of stroke associated with TCM use.

Characteristic	Non-Users *n* = 17,015	TCM Users *n* = 4912
Person-years of follow-up	68,527	22,927
All stroke		
No. of events	1764	523
Incidence rate/1000 person-years	25.7	22.8
Hazard ratio (95% confidence interval)		
Unadjusted	1	0.87 (0.79, 0.97)
Adjusted for age	1	0.91 (0.83, 1.01)
Multivariable adjusted *	1	0.93 (0.84, 1.03)
Ischemic stroke		
No. of events	1562	463
Incidence rate/1000 person-years	22.8	20.2
Hazard ratio (95% confidence interval)		
Unadjusted	1	0.88 (0.79, 0.98)
Adjusted for age	1	0.92 (0.82, 1.02)
Multivariable adjusted *	1	0.93 (0.83, 1.04)
Hemorrhagic stroke		
No. of events	224	64
Incidence rate/1000 person-years	3.3	2.8
Hazard ratio (95% confidence interval)		
Unadjusted	1	0.83 (0.62, 1.11)
Adjusted for age	1	0.87 (0.65, 1.16)
Multivariable adjusted *	1	0.89 (0.66, 1.19)

Abbreviations: TCM, traditional Chinese medicine. * The models were adjusted for age, sex, hypertension, hyperlipidemia, atrial fibrillation, ischemic heart disease, chronic kidney disease, and number of inpatient visits.

**Table 3:** Incidence rate and hazard ratio of incidence of stroke associated with TCM use.

Characteristic	Acupuncture *n* = 1094	Herbal Formulas *n* = 3627	Both *n* = 191
Person-years of follow-up	5141	17,020	765
All stroke			
No. of events	98	403	22
Incidence rate/1000 person-years	19.1	23.7	28.7
Hazard ratio (95% confidence interval)			
Unadjusted	0.73 (0.60–0.90)	0.91 (0.81–1.02)	1.10 (0.72–1.68)
Adjusted for age	0.76 (0.62–0.93)	0.95 (0.85–1.07)	1.19 (0.78–1.82)
Multivariable adjusted *	0.77 (0.63–0.95)	0.97 (0.86–1.08)	1.19 (0.78–1.82)
Ischemic stroke			
No. of events	91	353	19
Incidence rate/1000 person-years	17.7	20.7	24.8
Hazard ratio (95% confidence interval)			
Unadjusted	0.77 (0.62–0.95)	0.90 (0.80–1.02)	1.08 (0.68–1.69)
Adjusted for age	0.79 (0.64–0.98)	0.94 (0.84–1.06)	1.16 (0.74–1.83)
Multivariable adjusted *	0.81 (0.65–1.00)	0.96 (0.85–1.08)	1.16 (0.74–1.82)
Hemorrhagic stroke			
No. of events	9	52	3
Incidence rate/1000 person-years	1.8	3.1	3.9
Hazard ratio (95% confidence interval)			
Unadjusted	0.52 (0.27–1.02)	0.91 (0.66–1.24)	1.15 (0.37–3.62)
Adjusted for age	0.54 (0.28–1.06)	0.95 (0.69–1.30)	1.25 (0.40–3.93)
Multivariable adjusted *	0.56 (0.29–1.10)	0.96 (0.70–1.32)	1.31 (0.42–4.13)

Abbreviations: TCM, traditional Chinese medicine. * The models were adjusted for age, sex, hypertension, hyperlipidemia, atrial fibrillation, ischemic heart disease, chronic kidney disease, and number of inpatient visits.

**Table 4:** Adjusted hazard ratios of stroke in relation to various classes of prescribed Chinese herbal medicines.

Formula Classified by Actions on the Body	Ischemic Stroke			Hemorrhagic Stroke		
	No. of Events	Incidence *	Hazard Ratio ^†^ (95% CI)	No. of Events	Incidence *	Hazard Ratio ^†^ (95% CI)
Tonic formulas	87	23.0	1.09 (0.88–1.36)	8	2.1	0.67 (0.33–1.37)
Heat-clearing formulas	48	20.1	0.96 (0.72–1.28)	7	2.9	0.98 (0.46–2.09)
Blood-regulating formulas	55	21.9	1.05 (0.80–1.38)	11	4.4	1.49 (0.81–2.74)
Exterior-releasing formulas	50	20.5	0.99 (0.75–1.31)	8	3.3	1.11 (0.55–2.26)
Wind-dampness-dispelling formulas	62	25.5	1.19 (0.92–1.54)	13	5.3	1.80 (1.02–3.16)
Dryness-relieving formulas	44	18.7	0.83 (0.61–1.12)	7	3.0	0.91 (0.43–1.93)
Harmonizing formulas	38	17.9	0.86 (0.63–1.19)	9	4.2	1.46 (0.75–2.85)
Phlegm-dispelling formulas	33	19.8	0.94 (0.66–1.33)	10	6.0	2.02 (1.07–3.83)
Dampness-dispelling formulas	35	24.5	1.14 (0.81–1.59)	8	5.6	1.83 (0.90–3.72)
Downward draining formulas	29	17.7	0.84 (0.58–1.21)	5	3.0	1.01 (0.42–2.45)
Exterior- and interior-releasing formulas	22	13.8	0.66 (0.44–1.01)	6	3.8	1.30 (0.57–2.93)
Sedative formulas	37	25.4	1.19 (0.86–1.65)	7	4.8	1.58 (0.74–3.36)
Cold-dispelling formulas	14	19.4	0.91 (0.54–1.55)	2	2.8	0.91 (0.22–3.65)
Qi-regulating formulas	10	16.2	0.72 (0.39–1.34)	2	3.2	0.96 (0.24–3.86)
Formulas that treat abscesses and sores	4	20.2	0.91 (0.34–2.43)	2	10.1	3.13 (0.77–12.7)
Summer heat-clearing formulas	18	23.4	1.04 (0.65–1.65)	4	5.2	1.65 (0.61–4.44)
Astringent formulas	6	17.1	0.73 (0.33–1.64)	2	5.7	1.64 (0.41–6.64)
Purgative formulas	5	21.1	0.89 (0.37–2.13)	0	0	
Formulas for menstruation and childbirth	0	0		0	0	

* per 100,000 person-years. ^†^ The models were adjusted for age, sex, hypertension, hyperlipidemia, atrial fibrillation, ischemic heart disease, chronic kidney disease, and number of inpatient visits.

## Supplementary Materials

The following are available online at http://www.mdpi.com/1660-4601/15/6/1267/s1, Table S1: The diagnosis codes of ICD-9-CM for patient cohort, exclusion, comorbidity, and outcome; Table S2: Sensitivity analysis for hazard ratio of stroke in relation to TCM use defined as at least two claims of TCM outpatient visits in one year after the index date. Figure S1: Probability of free-from-stroke for TCM users and non-users.
