# Supplementary material for: Traditional Chinese Medical Care and Incidence of Stroke in Elderly Patients Treated with Antidiabetic Medications
Source: Int J Environ Res Public Health. 2018 Jun 15;15(6):1267. doi: 10.3390/ijerph15061267 (PMC6024911; doi:10.3390/ijerph15061267)

Supplemental Table S1. The diagnosis codes of ICD-9-CM for patient cohort, exclusion, comorbidity and outcome

Categories	Diseases	Diagnosis codes of ICD-9-CM
Patient cohort	Diabetes	250.xx
Exclusion	Stroke	430.xx-438.xx
Comorbidity	Ischemic heart disease	410.xx-414.xx
Comorbidity	Hypertension	401.xx-405.xx
Comorbidity	Hyperlipidemia	272.xx
Comorbidity	Atrial Fibrillation	427.3x
Comorbidity	Cancer	140.xx-208.xx
Comorbidity	Chronic kidney disease	585.xx
Outcome	Stroke	430.xx-437.xx (hemorrhagic, 430.xx-432.xx; ischemic, 433.xx-437.xx)

ICD-9-CM indicates International Classification of Diseases, Ninth Revision, Clinical Modification.

Int. J. Environ. Res. Public Health **2018**, 15, 1267; doi:10.3390/ijerph15061267 www.mdpi.com/journal/ijerph

Supplemental Table S2. Sensitivity analysis for hazard ratio of stroke in relation to TCM use defined as at least two claims for TCM outpatient visit in one year after index date

	Non-users (n=15379)	TCM users (n=1636)
Person-years of follow-up	82363	9090
All stroke		
No. of events	2077	210
Incidence rate / 1000 person-years	25.2	23.1
Unadjusted	1	0.94 (0.81-1.09)
Adjusted for age	1	0.97 (0.84-1.12)
Multivariable adjusted*	1	0.98 (0.85-1.13)
Ischemic stroke		
No. of events	1836	189
Incidence rate / 1000 person-years	22.3	20.8
Hazard ratio (95% confidence interval)		
Unadjusted	1	0.96 (0.83-1.12)
Adjusted for age	1	0.99 (0.85-1.15)
Multivariable adjusted*	1	1.00 (0.86-1.16)
Hemorrhagic stroke		
No. of events	266	22
Incidence rate / 1000 person-years	3.2	2.4
Hazard ratio (95% confidence interval)		
Unadjusted	1	0.76 (0.49-1.17)
Adjusted for age	1	0.78 (0.51-1.21)
Multivariable adjusted*	1	0.79 (0.51-1.23)

Abbreviations: CI, confidence interval; TCM, traditional Chinese medicine.

*Models were adjusted for age, sex, hypertension, hyperlipidemia, atrial fibrillation, ischemic heart disease, chronic kidney disease and number of inpatient visit.

Figure S1. Probability of free from stroke for TCM users and non-users

(a) All stroke

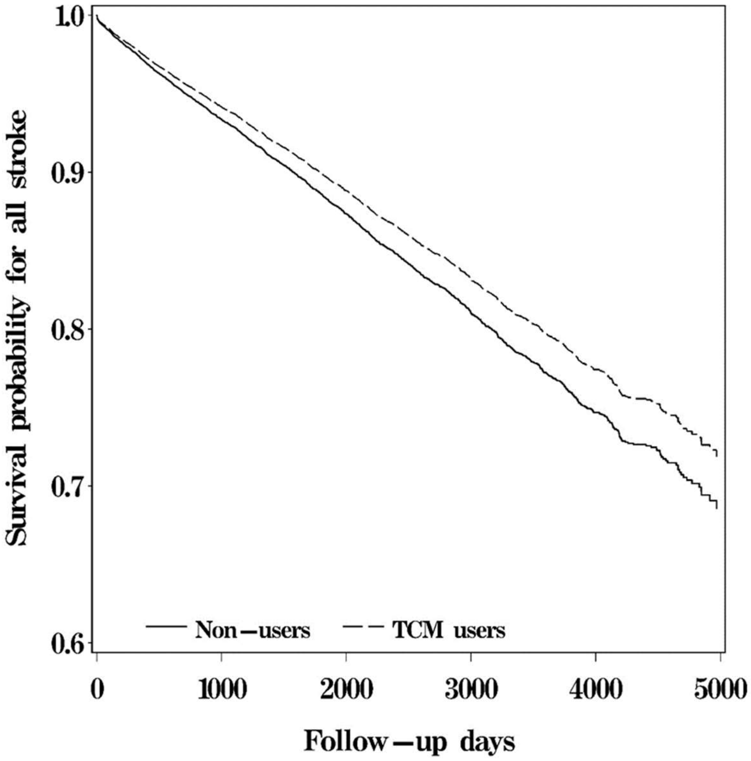

(b) Ischemic stroke

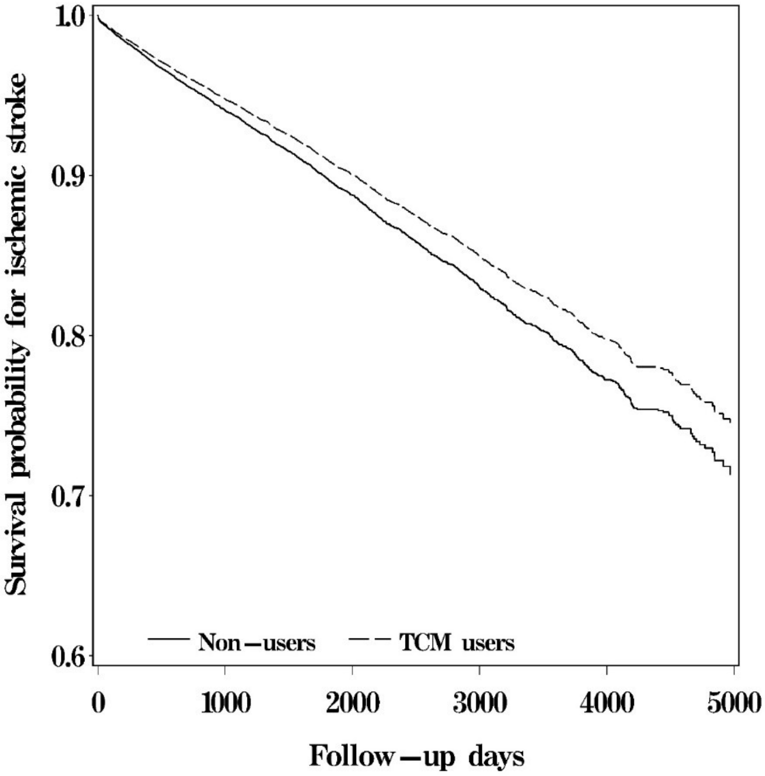

Int. J. Environ. Res. Public Health **2018**, 15, 1267; doi:10.3390/ijerph15061267 www.mdpi.com/journal/ijerph

(c) Hemorrhagic stroke

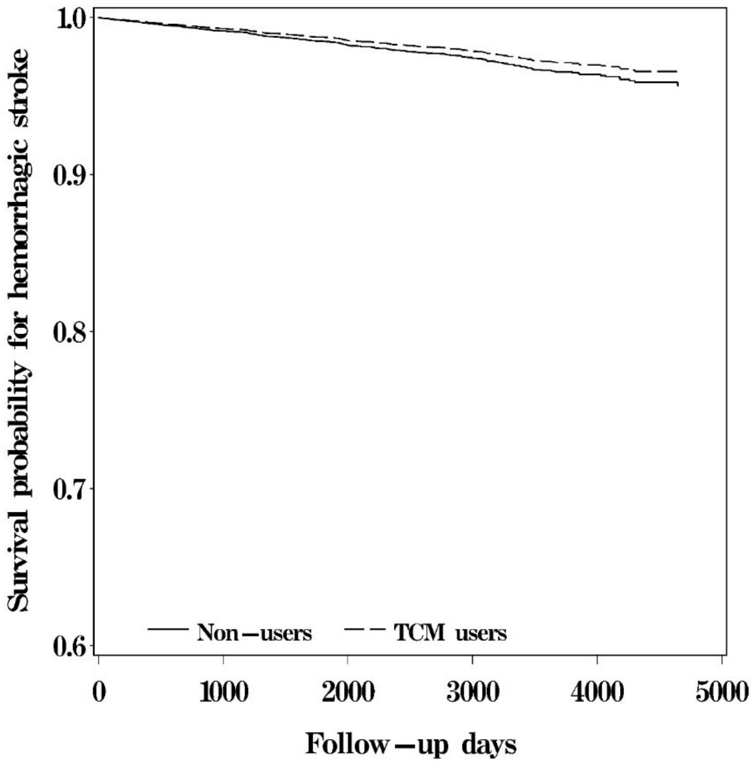

Supplement: Supplementary file 1 [file ijerph-15-01267-s001.zip › ijerph-306796-SI.pdf]
